# Ethical and legal aspects concerning robotic surgery in Brazil

**DOI:** 10.1590/0100-6991e-20243787-en

**Published:** 2024-12-02

**Authors:** EDUARDO NEUBARTH TRINDADE, ESTÉFANI LUISE FERNANDES TEIXEIRA, VANESSA SCHIMIDT BORTOLIN, LUCAS DOS SANTOS DIFANTE, MANOEL ROBERTO MACIEL TRINDADE

**Affiliations:** 1 - Hospital de Clínicas de Porto Alegre(HCPA) / Universidade Federal do RIO Grande do Sul (UFRGS), Serviço de Cirurgia Digestiva/Departamento de Cirurgia - Porto Alegre - RS - Brasil; 2 - Conselho Regional de Medicina do Rio Grande do Sul - Porto Alegre - RS - Brasil; 3 - Ordem dos Advogados do Brasil, Comissão Especial do Direito à Saúde - Porto Alegre - RS - Brasil

**Keywords:** Telesurgery, Robotic Surgical Procedures, Liability, Legal, Ethics, Medical, Telecirurgia, Procedimentos Cirúrgicos Robóticos, Responsabilidade Civil, Ética Médica

## Abstract

Robotic surgery is a technological milestone that directly impacts the provision of healthcare services. Procedures that utilize robotics are continuously being developed. In this context, it is important to analyze the distribution of ethical and civil liability among doctors, hospitals, and suppliers of surgical equipment in cases of alleged medical errors or adverse events that may harm patients. This review aims to examine the civil and ethical liability of the parties involved in robotic surgeries, as well as possible means to avoid legal complications related to these procedures concerning the Medical Councils and the judicial system.

## RESUMO

A cirurgia robótica é um marco tecnológico que impacta diretamente na prestação de serviços de saúde. Os procedimentos que utilizam robôs estão em constante desenvolvimento. Neste contexto, é importante analisar a distribuição da responsabilidade ética e civil envolvendo médicos, hospitais e fornecedores de equipamento cirúrgico, em casos de suspeição de erro médico ou eventos adversos que causem dano ao paciente. Esta revisão visa examinar a responsabilidade civil e ética dos atores envolvidos em cirurgias robóticas, bem como possíveis meios de evitar complicações legais relacionadas a esses procedimentos juntos aos Conselhos de Medicina e a Justiça Comum.

## INTRODUCTION

Technological advances in medicine have important impacts on the provision of health services. Robotic surgery has become popular in recent decades and is already used in practically all surgical specialties^1^. The da Vinci^®^ (Intuitive Surgical, CA) surgical system, introduced in the late 1990s, received first approval by the United States Food and Drug Administration (FDA) on July 11, 2000, for use in laparoscopic procedures. In 2009, the launch of the system’s third generation, da Vinci Si^®^, improved surgeons’ training and experience, expanding the capabilities of minimally invasive robotic surgery worldwide. Among the advantages it offers are the ability to provide virtual information, accuracy in spatial and geometric resolution, greater dexterity, and faster maneuverability. In addition, the ability to operate without fatigue ensures consistent and stable movements, factors that can bring potential advantages, such as reducing blood loss and postoperative recovery time, which may result in better clinical outcomes for patients^2^ ^,^ ^3^.

Despite these advances, robotic surgery is still under development and involves new risks, different from those found in laparotomy or laparoscopic procedures. In the initial stages of implementation, its risks were mainly related to the delay in response between the surgeon’s movements and their replication by the robot (time delay), insufficient training of physicians and staff, and defects in the robotic system itself^4^. Between 2000 and 2013, 10,624 adverse events were reported, 8,061 due to robot malfunction, 1,391 due to patient injuries, and 144 due to patient death5. Currently, defects due to improper equipment use are also reported, mainly friction and collision of the robotic arms inside the abdominal cavity or during their insertion into the trocars.

Thus, there is a need to understand the ethical and legal implications of this practice, a topic that has not yet been fully explored. This article presents a brief overview of recent technologies in the health area, with emphasis on robotic surgery and telesurgery, their regulation in Brazil, and the Certification Guidelines in Robotic Surgery by the Brazilian College of Surgeons (CBC). The work investigates ways to decrease human errors (especially medical errors) and mitigate harm in robotic surgery adverse events. We will discuss the distribution of civil liability in cases patient harm, as well as the wide range of situations and legal responsibilities existing in medical errors or adverse events when operating with the robot, and even the structure of the hospital.

### Regulation of robotic surgery in Brazil

In Brazil, the regulation of robotic surgery occurred through CFM Resolution 2,311/20226, when the Federal Council of Medicine defined it as “a surgical treatment modality to be used by a minimally invasive route, open or combined, for the treatment of diseases in which its efficacy and safety have already been proven”, establishing the practice as a “highly complex procedure” (article 1 and paragraph 1). The regulation requires that robotic surgeries can only be performed by a physician with a Specialist Qualification Title (SQT) in the surgical area related to the procedure and training in robotic surgery during residency or in specific instruction (article 3 and article 1). One should always remember that robotic surgery is not a new specialty or area of expertise itself, but a new technological tool available to surgeons.

The CFM Resolution also provides for a main surgeon, responsible for direct patient care regarding diagnosis, choice of technique and intraoperative and postoperative complications (article 3, paragraph 4), and an instructor-surgeon, responsible for guiding the handling of the robot and evaluating the competence of the main surgeon, not participating directly in patient care (article 4). For the main surgeon to be able to perform robotic surgeries without the participation of an instructor-surgeon, they must have completed specific training and performed a minimum of 10 robotic surgeries. Paragraph 2 of article 1 of the CFM Resolution provides for the need for a specific Informed Consent Form (ICF) for the procedure, with clarifications on the risks and benefits of the procedure. 

The regulation requires several other things, including efficient and redundant communication bandwidth, stability in the supply of electricity, and security against computer viruses or system invasion by hackers. In addition, robotic surgeries must be performed in hospitals that meet the requirements of high complexity, that is, they must have technical conditions, physical facilities, equipment, and adequate support services to provide specialized care to patients undergoing robot-assisted surgeries.

### Telesurgery

Advancing on the subject, the same CFM regulation also conceptualized and standardized robotic surgery performed at a distance (article 6) - robotic telesurgery -, defining it as the performance of a surgical procedure using robotic equipment, mediated by safe interactive technologies. In other words, in robotic telesurgery, the surgeon is geographically distant from the patient. In these cases, there is also a need for a surgeon and an assistant surgeon present, to assist the patient in case of robot malfunction or technological interruptions.

It is interesting to note that even before the first regulation by the CFM, on September 9, 2020, the Brazilian College of Surgeons had already published the Certification Guidelines for Robotic Surgery^7^, with the objective of proposing a minimum curriculum for the development of proficiency in robotic surgical procedures. This initiative was important because it followed technological evolution to ensure adequate training for physicians and, therefore, patient safety.

Despite all the benefits and the fact that we already have regulations on the subject, there are reports of adverse events occurring during robot-assisted surgeries, in addition to dozens of recalls of defective robotic instruments4, bringing to light the need to reflect on the responsibilities involved in this type of procedure.

### Physician’s civil liability in robotic surgery

Civil liability is an extremely relevant topic for all legal science and, to the extent that it interacts with medical activity, it requires an attentive and specialized look at the peculiarities of this area and the doctor-patient relationship8. Considering the technological innovations and the plurality of subjects involved in robotic procedures, the determination of how civil liability is distributed in adverse events is challenging. 

Miguel Kfouri systematized a methodology for analyzing the event causing the damage, characterizing its genesis as a medical, paramedical, or extramedical service. It is also necessary to verify the causal link between the conduct and the damage suffered^9^.

According to this methodology, therefore, the first assessed conduct is the physician’s, to verify the occurrence of fault stricto sensu on the part of the professional. In this case, when the damage has occurred in a medical service (acts performed exclusively by medical professionals), the analysis of liability will be subjective, depending on the proof of malpractice, recklessness or negligence^10^. 

Currently, the prevailing tendency in Brazilian Courts is to understand that medical activity is a consumer relationship and, therefore, subject to the precepts of the Consumer Protection Code (CDC). Thus, the legislation applied in cases that analyze problems in medical services is primarily the CDC, and only subsidiarily the Civil Code (CC), considering the technical and informational hyposufficiency of the patient seen as a consumer. However, even applying the consumer regulations, the analysis of the medical professional liability remains subjective, under the terms of article 14, paragraph 4 of the CDC^11^ - that is, it does not dispense with the presence of malpractice, recklessness, or negligence. 

Even so, especially in procedures involving different participants, such as robotic surgery, the fear of litigation in case of surgical complications can reduce the availability of instructor-surgeons, also called proctors^1^2. We should note, however, that under the terms of Res. CFM. 2,311/2022, the responsibility of the proctor is expressly linked to guidance in the handling of the robot and in the evaluation of the competence of the main surgeon (article 4). In addition, the glossary that accompanies the CFM regulation provides that, during the performance of robotic surgery, the instructor-surgeon will guide the main surgeon in the handling of the robot, including the console and robotic instruments, “their responsibility not being to participate in the surgical indication, the choice of the surgical technique, or even the direct care to the patient in the intraoperative or postoperative period”. However, a topic that cannot be neglected is the figure of the “ghost” surgeon, whose practice has grown in the context of robotic surgery and is often not communicated to patients. This practice, in which the proctor assumes the main role in the surgical act, raises important ethical and legal questions, making it necessary to consider the responsibility of both the main and the “ghost” surgeons. According to article 5 of Res. CFM. 2,311/2022, the hospital’s technical director must ensure competence and training of both the main and instructor surgeons, including requiring the identification of all team members in the surgical description. Therefore, one cannot forget the possible responsibility of the proctor, specifically during the intraoperative period, making an analogy to the case of the resident-preceptor relationship.

The responsibility of medical professionals involved in robotic surgery and telesurgery should be analyzed according to the specific characteristics of each modality, considering the complexity and risks inherent to these technologies. In robotic surgery, the primary responsibility falls on the surgeon operating the robot. This professional must have specialized training and is responsible for all stages of the procedure, from the decision to use robotic surgery to the supervision of the assistant team. In the event of equipment failure, there may be joint liability between the robot manufacturer and the hospital for any damage caused to the patient.

In robotic telesurgery, in addition to the technical and operational responsibilities like those of robotic surgery, there is an increase in complexity due to the physical distance between the operating surgeon and the patient. The remote surgeon is responsible for ensuring that the communication technology works properly, but the presence of a local team at the surgical site is crucial for performing emergency interventions. 

Having made these reservations regarding the different surgery modalities, if the subjective fault of the physician is recognized, the hospital will be jointly and severally liable, under the terms of article 14 of the CDC and articles 186 and 951 of the CC10. It is important to highlight, however, that if the physician does not have a direct link with the hospital, the institution may or may not respond in a joint manner^11^, depending on the specificities of the case. 

It is also possible that the harmful event was a consequence of a risk associated with the technology itself, without any professional or equipment failure. In this case, it will be essential to prove that the patient signed the informed consent about that specific risk arising from the use of technology. The triggering event for compensation in cases of violation of the duty to inform will not be the damage itself, in isolation, but the failure in the duty to inform8. It may also happen that the damage has as a triggering event a failure in the paramedical service, that is, in the conduct of nurses in regulating the robot or sterilizing robotic instruments. In this case, the hospital will be objectively liable for the acts of its representatives, also under the terms of article 14 of the CDC. 

On the other hand, if it is proven that the damage was caused due to a defect in the robot’s own software or one of its parts, or even by insufficient information about its use/risks, the manufacturer will be liable regardless of the existence of fault (strict liability). In this regard, under the concept of suppliers contained in article 3 of the CDC, the hospital will be considered part of the consumption chain and may be held jointly and severally liable to the manufacturer of the robotic equipment. 

The damage may also have resulted from what Kfouri’s classification calls an extra-medical service9, that is, from an insufficient or even non-existent hospital policy for training professionals in the use of robots. In this case, also under the terms of article 14 of the CDC, the hospital will respond objectively. In this regard, it is worth noting that the hospital entity often invests large financial resources in the acquisition of the surgeon robot. It then expects a quick economic return, which can sometimes be incompatible with the adequate training of the team for its use. At this point, and in all other cases on civil liability in the case of adverse events, the Certification Guidelines for Robotic Surgery, issued in 2020 by the Brazilian College of Surgeons (CBC), are essential. 

The training required to obtain the certification recommended by the CBC has great potential to reduce not only human errors (surgeon error), but also to reduce or even avoid damage in cases of adverse events arising from the robot’s own operation, as it involves adequate technical knowledge regarding the configuration of the robotic platform and “troubleshooting and system emergency”. This way, if the recommendations contained in the document prepared by experts in the field are adopted, physicians involved in both robotic surgery and telesurgery will be more prepared for the correct use of the equipment, to configure the robotic platform and its systems, to correctly position the surgical platform for different surgical procedures, to analyze possible problems that may affect the configuration and proper fitting and unfitting of the platform robotic surgical system, and to solve emergency situations presented by the system.

The suggested training, whose certification will be granted by the Brazilian Medical Association (AMB), has several stages: basic training with a presentation of the robotic system; theoretical-practical training on the robotic platform through face-to-face observation inside the operating room; pre-clinical stage for the development of psychomotor skills with surgical simulation (through virtual reality or in organic models); and clinical stage, consisting of clinical training under tutoring, assisting in robotic procedures, and performing them under supervision. 

Annex II of CFM Resolution 2,311/2022 also provides for phases of specific training in robotic surgery. Although leaner, in some respects it resembles the document prepared by the Brazilian College of Surgeons, thus reinforcing the idea that the training of professionals is an essential measure to reducing or even avoiding professional errors or harm to patients in cases of adverse events or failure of the robotic equipment.

## FINAL CONSIDERATIONS

We analyzed the civil liability of the subjects involved in robotic surgery in the event of medical error or adverse events, concluding that the personal liability of the physician will always be subjective, not dispensing with the recognition of the occurrence of malpractice, recklessness, or negligence. In the event of recognition of the medical professional’s fault, if there is a link with the hospital, the institution will respond in a joint and several manner. 

In addition, if the damage to the patient occurred due to a risk inherent to the procedure, in the absence of any medical error or adverse event, the doctor or the hospital may be held liable if an Informed Consent Form (ICF) has not been collected, which has adequately informed the patient about the risks related to the use of the technology. We also concluded that, in the event of failure of the robotic equipment, the supplier responds objectively, and the hospital is also considered a supplier of the consumption chain, responding objectively alongside the manufacturer. 

We identified three indispensable factors for the safe performance of robot-assisted procedures, i.e., adequate hospital structure, training and accreditation of the professionals involved, and specific ICF for the use of the technology. The Guidelines developed by the CBC, if properly observed, have great potential for reducing surgeon failures and damage in the event of adverse events, as they require the medical professionals involved in the procedure to have in-depth technical knowledge about the operation of the robotic equipment and for the solution of emergency problems in the system.

## References

[1] Leal Ghezzi T, Campos Corleta O (2016). 30 Years of Robotic Surgery. World J Surg.

[2] Lane T (2018). A short history of robotic surgery. Ann R Coll Surg Engl.

[3] Muaddi H, Me Hafid, Wj Choi, Lillie E, Lc De Mestra, Nathens A (2021). Clinical Outcomes of Robotic Surgery Compared to Conventional Surgical Approaches (Laparoscopic or Open) A System-atic Overview of Reviews Ann. Surg.

[4] Patel V, Saikali S, Moschovas MC (2024). Technical and ethical considerations in telesurgery. J Robot Surg.

[5] Nogaroli R (2022). Responsabilidade civil médica e consentimento do paciente nas cirurgias robóticas realizadas à distância (telecirurgias). Telemedicina: Desafios Éticos e Regulatórios (.

[6] Conselho Federal de Medicina Resolução CFM n. 2.311 de 28 de março de 2022. Regulamenta a cirurgia robótica no Brasil.

[7] Colégio Brasileiro de Cirurgiões Diretrizes de Certificação em Cirurgia Robótica 2020.

[8] Muniz G, Barbosa C (2024). A responsabilidade civil médica diante do inadimplemento do dever de informação na cirurgia robótica à luz da resolução do CFM n.º 2.311/22. Revista Eletrônica da OAB Joinville.

[9] Kfouri M, Nogaroli R (2020). Estudo comparatístico da responsabilidade civil do médico, hospital e fabricante na cirurgia assistida por robô..

[10] Dantas E (2022). Direito médico.

[11] Mota M, Farias C, Tavares T, Santana A (2023). Os limites da responsabilidade Civil do médico em cirurgias robóticas. CGCHS.

[12] Lee YL, Kilic G, Phelps JY (2012). Liability exposure for surgical robotics instructors. J Minim Invasive Gynecol.

