# Erratum

**DOI:** 10.1590/0100-6991e-20253787errata

**Published:** 2025-03-11

The article “Ethical and legal aspects concerning robotic surgery in Brazil”, DOI number: 10.1590/0100-6991e-20243787-en, published in the Journal of the Brazilian College of Surgeons 51:e20243787.

Where it is:

Authorship:

VANESSA SCHIMIDT BORTOLIN

https://orcid.org/0000-0002-3200-4845

It should be:

Authorship:

VANESSA SCHMIDT BORTOLINI

https://orcid.org/0000-0002-3200-4845

